# It’s Not Your Parents Long-Term Services : A 30 Year Longitudinal Study of Ohio’s System

**DOI:** 10.1093/geroni/igaf122.2692

**Published:** 2025-12-31

**Authors:** Robert Applebaum, Matt Nelson, John Bowblis

**Affiliations:** Miami University, Oxford, Ohio, United States; Miami University, Oxford, Ohio, United States; Miami University, Oxford, Ohio, United States

## Abstract

Using data from a longitudinal study of Ohio’s long-term services and supports system this paper documents the monumental system changes occurring over the last three decades. The study is based on a biennial survey of nursing homes and residential care facilities in the state (92% survey response rate) and records data on participants in Ohio’s home and community-based waiver program. Findings from the study document an almost un-imaginal change in how long-term services are provided. As an example, Ohio with a politically powerful nursing home industry, was widely dependent on nursing home care for older people age 60 and older needing long-term services in the early 1990’s. In 1993, nine in ten older people on Medicaid needing long-term services received such support in the nursing home setting. In 2023, with the expansion of home and community-based services, that proportion had dropped to four in ten. During this time period that state’s population age 85 and older increased by more than 100,000, but the number of nursing home beds actually dropped by 12%. Additionally, occupancy rate declined from 92% in 1992 to 79% in 2023. The assisted living industry (licensed as residential care facilities in Ohio) grew dramatically during this time period, increasing from less than 200 in 1992 to more than 800 in 2023. The number of short-term admissions to nursing homes also increased dramatically, going from 30,000 in 1993 to 126,500 in 2023. The paper documents these and other changes and discusses what these changes mean for the future.

